# The Use of the Key

**Published:** 1848-10

**Authors:** J. Taft

**Affiliations:** Xenia, O.


### THE USE OF THE KEY.

By Dr. J. Taft, *Xenia, O.*

Frequently I am asked, why I do not use the key for extracting teeth; and occasionally, I have been thus interrogated by physicians of some eminence in their profession; and sometimes the interrogatory has had in its compound a few touches of sarcasm; "Why is it," they say, "that you abandon," for I never use it, "and condemn the tooth key," the use of which has been sanctioned, and continued from time immemorial?" The merit of anything is not to be estimated by its long sanctioned usage. Why is it that the key is so highly esteemed by a great majority of physicians, and by quite a number of dentists too? Is it because it never fails them in an operation? It cannot be, for facts show it to be far otherwise. Many take exceptions to our objections against the key, not from any analysis of our objections either, but because, in their estimation, it is the best instrument for extracting teeth, when perhaps they have never made trial of any other, except, it may be, a pair of old, rusty, ill-constructed forceps, properly adapted for no conceivable purpose. Never have I known any advocate of the key to advance its mechanical modus operandi, in support of its superiority over all other instruments; for if any one will take the task, and analyze its modus operandi, and this I will endeavor presently to do, he will at once be convinced of its inefficiency. And this inefficiency will be further apparent, when we consider the fact, that from thirty to fifty per cent., of all the efforts to extract teeth with this instrument prove abortive; and these unsuccessful attempts do not occur because the teeth so attempted are more difficult to remove than others, for we find such teeth as easily removed with the forceps, as others of the same class. But these failures result from the unfitness of the instrument, together with, oftentimes, a bungling operator. I have a number of objections to this instrument, which, for the most part, will be more easily comprehended after an analysis of its modus operandi. In an examination of this instrument, it is quite apparent that the principle of its power is the same in all the modifications that have been heretofore made upon it;* and so far as there is any objection to the *principle* of one form of key, the same objection stands against all its modifications, especially all those I have had an opportunity to examine. This instrument consists of the axis shaft, with its handle attached at one end, and at the other the bolster or fulcrum, and hook; the bolster is that part, having its base upon the gum, in the operation, by which the counteracting force is sustained; the hook is attached to the axis shaft, directly above the bolster, and starts off at a right angle with the vertical axis of the bolster, but curves downward till the point of the hook is almost, or quite as low as the base of the bolster; the point of this hook, in the operation, embraces the tooth at its neck, upon the side opposite the seat of the bolster. This hook, when properly constructed, embraces the tooth at the neck upon one side, and the bolster rests a little below the neck of the tooth upon the other side. When this instrument is applied to a tooth, the centre of the shaft is the axis of motion, but as force is applied to the handle of the instrument, the axis of motion is transferred from the shaft to the base of the bolster, it being a perfect axis of motion the moment it is permanently fixed upon the gum and alveolus. The shaft is no longer the axis of motion, but describes an arch about the base of the bolster. Now as a result of this motion, the direct line of force exerted upon the tooth by the instrument, is at an angle of from forty to fifty degrees with the axis of the tooth; and hence, it is at this angle that the tooth must be extracted, if at all.† The axis of power exerted by the in-

---

*Harras' Principles and Practice of Dental Surgery, p.333, 3d ed.

†Desirabode on Mechan. Dentistry, sec. 6 on the Key.

strument upon the tooth, is a direct line from the point of the hook to its attachment to the shaft of the instrument. And the line of this force, in regard to the tooth, has its termination below the neck of the tooth upon one side, and above the crown of the tooth at the opposite side. The angle formed by the line, or axis of power, with the axis of the tooth, is different in the different relative positions of the key to the tooth; if the key is placed upon an inferior molar, with the fulcrum on the inside, as directed by Dr. Harras,* the angle of the line of force with the axis of the tooth would be about forty degrees; but if placed upon the outside of the jaw, as directed by Fitch's Desirabode, and others, the angle contained by the line of power and axis of the tooth, would be sixty degrees or more. This axis, or line of power, is not changed by any form the hook may assume between these two points, viz: the point of the hook and its attachment. It may assume a regular curve, or an elliptical form, or be turned at a right angle, and yet the axis of power not be changed at all; the axis of power is only changed by changing the relative position of those two points. The increasing or diminishing the curve of the hook, is only to form a proper receptacle for the crown of a large or small tooth. This application of power to extract teeth, constitutes my first objection to the use of the key. The force is applied at too great an angle with the axis of the tooth; and hence in numerous instances the tooth is broken off, and if it comes away at all, the alveolus possessing but little elasticity and being set close about the tooth, suffers fracture to a greater or less extent, according to the angle at which the tooth is removed. This difficulty is somewhat increased by the divergence of the fangs. The power requisite to extract a tooth *with this application* is greatly increased, and hence to extract a tooth with the *key* is much more difficult than with an instrument of a different application, for the operator, and more painful for the patient.

Again, the bolster of the key is placed upon the gum, and in extracting, great pressure is exerted by it upon the gum and al-

---

*Harras' Principles and Practice of Dental Surgery, p.342, 3d ed.

veolus; and hence, the gum is always bruised, and frequently lacerated and cut in a cruel manner; and yet, strange to tell, there are persons familiar with this fact, who use the instrument, and advocate its use. The amount of pressure caused by the bolster of the variously constructed keys, differs but little; though it is but proper to remark, that in the opinion of the writer, the bolster with a broad base, and articulated to the axis shaft by a joint, would cause less pain to the patient by its pressure, and is much less liable to lacerate or cut the gum, than the small and permanent bolster. This is deduced from the fact that much more power is requisite to produce the same effect upon a large surface, than upon a small one. The small permanent bolster moving upon the gum with such force or pressure, greatly increases the probability of laceration of the gum, and fracture of the alveolus. In estimating the amount of pressure of the bolster upom the gum, it is only a relative proportion between this and the power requisite to extract any given tooth, that we wish to attain. I will say in the outset, that the pressure of the bolster upon the gum, and process is greater than the power requisite to extract the tooth. If the angle formed by the line between the *point* of the hook and its *attachment*, and the *vertical axis* of the bolster, was a rectangle, and these two lines of equal length, then the force exerted by the bolster upon the gum and process, would be equal to the power requisite to remove the tooth. In examination of the key we find neither of these to be the case. The vertical line of the bolster is much shorter than the line of the hook from its point to its attachment. And while this is the case, though the angle contained by these two lines was a right angle, which it never is, the pressure of the bolster, would be greater than the power at the point of the hook. But instead of the contained angle of these two lines being a right angle, it is an acute angle of from thirty-five to sixty degrees; never, according to my observation, being less than thirty-five, nor more than sixty degrees. Now what is the consequence of such a relative construction of parts? It must be an increase of power at the termination of the short line, or base of the bolster, and a decrease of power at the termination of the long line, or at the point of the hook. This is my second objection to the use of the key, viz: the increase of power exerted by the bolster upon the gum and process, over and above the power necessary to extract the tooth, and that too at the disadvantage of being removed at an angle of forty-five degrees, or more, with the axis of the tooth; and hence the injury sustained by the gum and process, under the influence of this instrument. It is worthy of remark here, in common to these two objections, that a tooth being removed from its socket, at an angle of forty-five degrees, would alone be sufficient in a great number of cases, to fracture the process; and I think it clearly shown that the action of the bolster tends to produce this result. These two forces then acting in concert to produce a fracture of the process, it will not appear strange that, as Desirabode remarks, it "*generally causes fracture of the alveolus.*" It is frequently remarked, to invalidate this objection, that the laceration of the gums, or even the fracture of the process, is a matter of no serious consequence, as the gum would heal, and the process would be absorbed away. This is true in part, but it does not meet the objection. The operation of extracting is severe enough at best, and what is the excuse for adding to this pain an hundred fold, and leaving the mouth in such a condition that it will be painful for weeks, or even months, as is oftentimes the case, after submitting to an almost intolerable operation.— And it *is true* that very serious consequences have resulted from fracture of the process; broken and dead pieces of the alveolus remaining attached to the gum, and producing inflammation and ulceration of the gum, and more than this, necrosis of the unbroken bone, serious consequences are sometimes involved in the latter. Another objection to the key is, that it is impossible to embrace a tooth as deep with the hook as with a well constructed pair of forceps. This may be deduced from two facts. In order that the hook seize firmly upon the tooth, its point must approach the tooth at an angle of about ninety degrees, and with this adaptation, it is impossible to apply the power as deep with the key, as with those instruments of a different construction and better adaptation. Again, that the hook may seize firmly upon the tooth, it must be, as it always is, very narrow at the seizing point, and then to give it strength, it is made much thicker near its point and upward, than it otherwise need be. This thickness of the hook, as well as the rectangular position of the point of the hook to the tooth, prevents the seizure or embrace being made as far below the neck of the tooth as is attainable with forceps. The deep embrace of a tooth in extracting, is an important consideration; because it requires less power, *thus applied*, to extract a tooth; the operator has better command of the instrument and the tooth; the tooth is not so liable to be broken; the process is not as liable to be fractured, and the pain is less to the patient. The three above mentioned, constitute *some* of my objections to the use of the key. There are a number of other objections equally important, about which I will say nothing for the present, though I may at another time, make them a matter of investigation. These above, however, partake more of an analytical character. It is for this reason that I have touched upon these in preference to others. While my views, upon the key and its modus operandi, remain as they are, I cannot but condemn it.

